# Australia's Divergent Legal Responses to Women Who Kill Their Abusive Partners

**DOI:** 10.1177/10778012231156154

**Published:** 2023-02-16

**Authors:** Caitlin Nash, Rachel Dioso-Villa

**Affiliations:** School of Criminology and Criminal Justice, 607305 Griffith Criminology Institute, Griffith University, Brisbane, QLD, Australia

**Keywords:** battered women, domestic violence, intimate partner homicide, law reform, self-defense

## Abstract

Concerns over the legal treatment of women who kill in response to domestic abuse have driven several jurisdictions to reform their homicide laws in recent years. This article examines how abused women are currently treated within the Australian legal system by analyzing homicide cases involving women prosecuted for killing an abusive partner in Australia from 2010 to 2020. The findings reveal the limitations of legal reforms to improve access to justice for abused women. Instead, there needs to be an increased focus toward pre-trial stages of criminal proceedings and to address persistent misconceptions and stereotypes associated with domestic abuse.

Intimate partner violence is the most prevalent and serious violence committed against women that can result in fatal consequences (Stöckl et al., 2013). While women are much more likely to be the victims of intimate partner homicides than the perpetrator, when women do kill a male intimate partner, it is most often out of fear and self-preservation in response to prolonged domestic abuse (Belknap et al. 2012; Domestic Violence Death Review Team, 2018; Morgan, 2002; Serran & Firestone, 2004). However, laws and legal systems have continually failed to recognize and accommodate the experiences of women who kill in the context of abuse. This issue has garnered substantial legal and academic attention in Australia and overseas, driving several jurisdictions to review and reform their homicide laws to improve access to defenses for abused women who kill (Fitz-Gibbon, 2022; Sheehy et al., 2012b). However, across Australia, these legal reforms have gone in divergent directions, resulting in different defenses and legal requirements for homicide (Crofts & Tyson, 2013; Fitz-Gibbon & Stubbs, 2012).

Given the divergent law reform activity in recent years, this study collected and examined 69 cases of abused women prosecuted for killing intimate male partners in Australia from 2010 to 2020. The aim was to examine the current legal outcomes of women prosecuted for killing an abusive male partner in Australia, and to consider how the different approaches adopted have impacted their legal position. Such findings can allow other criminal justice systems to learn from the divergent reform experiences, both in Australia and overseas. This can provide important policy implications for further reform efforts to improve the legal treatment of abused women who kill.

## Legal Barriers for Women Who Kill an Abusive Partner

The inability of the law to accommodate women who kill in the context of domestic abuse has been extensively criticized in adversarial criminal justice systems. A common criticism is the gender-biased operation of homicide laws; as defenses to homicide have been developed around male experiences of violence, their interpretation and application have largely excluded abused women from accessing relevant defenses (e.g., see Fitz-Gibbon & Vannier, 2017; Hopkins & Easteal, 2010; Mechanic, 2023). For instance, while the traditional association of self-defense as a one-off spontaneous encounter has comfortably accommodated men killing other men of relatively equal strength, it has made it difficult for women who kill in response to prolonged domestic abuse to successfully argue the defense (Victorian Law Reform Commission [VLRC], 2004). Due to discrepancies in size and experience of abuse, women who kill an abusive partner may do so in non-confrontational situations when the threat of harm is not immediate, such as when their partner is asleep or has their back turned (Braun, 2017; Hopkins et al., 2018; VLRC, 2004). Abused women also frequently use a weapon to protect themselves and, in some cases, enlist the assistance of others to kill their violent partners (VLRC, 2004). As a result, their actions can often be interpreted as “disproportionate” in the immediate circumstances and consequently considered “unreasonable” (Kirkwood et al., 2013, p. 6).

As such, while self-defense is universally acknowledged to be the preferable defense for those who kill in response to prolonged abuse, abused women are not generally successful in raising self-defense, particularly those who kill in non-traditional self-defense scenarios (Sheehy et al., 2012a; Tarrant, 2018; Tyson et al., 2017). In their review of homicide cases involving abused women from 2000 to 2010 in Australia, Canada and New Zealand, Sheehy et al. (2012a) found that of the 67 Australia cases identified, only 11 (16%) involved women being acquitted on the basis of self-defense, with only three of these cases involving non-confrontational circumstances when the threat of harm was not imminent. In Canada, there has only been one acquittal involving non-traditional self-defense circumstances, while none have been documented in New Zealand (Tolmie, 2015).

Abused women are also hindered by misconceptions and inaccurate understandings of the nature and dynamics of domestic abuse, with women often asked why they did not just leave the relationship (Douglas et al., 2020; Hopkins et al., 2018; Mechanic, 2023; Tarrant et al., 2019; Tyson et al., 2017). While expert evidence on battered woman syndrome (BWS) was originally introduced as a defense strategy in Australia, Canada, and the United States to help explain why a woman's resort to lethal violence may have been reasonable in the context of abuse, it has been extensively criticized for portraying their actions as the result of an individual deficit or pathology rather than a rational and reasonable response to serious domestic violence (Bradfield, 2002; Schneider, 2000; Stubbs & Tolmie, 1999). Generally presented by psychologists or psychiatrists, BWS evidence tends to pathologize women's responses to domestic abuse, ultimately excusing rather than justifying the offence (Butler, 2016; Tarrant et al., 2019; Tolmie et al., 2018).

BWS is also heavily criticized for reinforcing narrow stereotypes of what constitutes a “real” abused woman (Ferraro, 2003, p. 110; see also Rothenberg, 2003; Shaffer, 1997; Weare, 2013). For instance, the key concept of learned helplessness supports the myth and misconception that abused women are victimized, passive, helpless and compliant (Stubbs & Tolmie, 2005; Tarrant et al., 2019). However, research has found that women who do not conform to this narrow stereotype are treated more punitively, including women who fight back, experience problems with alcohol or drug abuse, have a prior criminal history, or demonstrate autonomous behavior in other spheres of her life (Delgado-Alvarez & Sanchez-Prada, 2021; Douglas, 2012; Fitz-Gibbon & Vannier, 2017; Goodmark, 2008). This can create a particular barrier for Indigenous women and women of color who are excluded from stereotypical constructions of a “real” victim that typically reflect white, middle-class norms (Larance et al., 2019; Stubbs, & Tolmie, 2008).

To provide a better understanding on the social realities of living within an abusive relationship, feminist scholars have called for a shift away from BWS evidence toward the use of social context evidence (Bradfield, 2002; Butler, 2016; Douglas, 2015; Terrance et al., 2012) or evidence on coercive control (Midson, 2016; Tyson, 2020). Rather than focusing on the individual's psychology, such evidence is intended to convey the wider social context in which the homicide took place and the objectively dangerous nature of serious domestic violence. However, scholars have highlighted the challenges and difficulties abused women have faced in having such evidence admitted in Australia and overseas (Butler, 2016; Chin, 2020; Sheehy, 2018; Tarrant et al., 2019).

Lastly, research in Canada, New Zealand, and Australia shows that abused women charged with murder are under considerable pressure to plead guilty to manslaughter rather than argue self-defense at trial and risk a murder conviction (Sheehy, 2014; Sheehy et al., 2012a; Stubbs & Tolmie, 2008; Tyson et al., 2017). In some Australian jurisdictions, there are mandatory minimum sentences for murder, with Queensland, the Northern Territory, South Australia retaining mandatory life imprisonment. This can provide strong incentives for plea bargaining as the stakes for a murder conviction can be “unbearably high” for many abused women (Stubbs & Tomie, 2005, p. 202). Research in Australia and Canada has found that the “overwhelmingly” majority of abused women have traditionally been charged with murder, even though in most of the cases the prosecution is willing to accept a plea to a lesser offence, with pleading guilty to manslaughter being the most common legal outcome for abused women (Sheehy, 2014; Sheehy et al., 2012a, p. 386). This situation has been described as abused women being “overcharged,” with research finding that many women who plead guilty to manslaughter demonstrated strong defensive elements and could have been acquitted based on self-defense had they gone to trial (Kirkwood et al., 2013; Sheehy, 2014; Sheehy et al., 2012a).

## Legal Reforms: Improving Access to Defenses to Homicide

Concerns over the legal position of abused women has animated numerous law reforms in both Australia and overseas to improve access to defenses to homicide for women who kill in the context of abuse (Fitz-Gibbon, 2022; Sheehy et al., 2012b). However, within Australia, each jurisdiction has taken a different approach to amending their homicide laws. Table 1 outlines the legal reforms that have been implemented throughout Australia in recent years, with each briefly described below.

**Table 1.** Recent Legal Developments to Homicide Laws in Australia by Jurisdiction and the Year They Were Introduced.

Changes	Vic	WA	NSW	Qld	SA	NT	ACT	Tas
Expanding self-defense								
Removed requirement for imminence	2005	2008						
Legislated social context evidence	2005	2020		1998	2020			
Introduced jury directions	2014	2020			2020			
Amending partial defenses								
Introduced a specialized defense				2010				
Abolished provocation	2005	2008			2020			2003
Retained provocation			2014	2011		2006	2003	
Introduced excessive self-defense^a^	2005	2008	2001		1997			
Abolished excessive self-defense^a^	2014							
Provides no partial defenses	2014							2003

^a^ *Note.* Also includes Victoria's offence of defensive homicide.

### Expanding self-defense

Common law jurisdictions including Australia, England and Wales, Canada, and New Zealand have all introduced new legislation amending the law of self-defense to better accommodate the circumstances in which women may kill an abusive partner (Fitz-Gibbon, 2022; Sheehy et al., 2012b). In Australia, the common law test of self-defense is set out in *Zecevic v. DPP* (1987) and requires that the accused honestly believes that their actions were necessary in self-defense and that there were reasonable grounds for this belief. While the common law test no longer requires a threat to be imminent, or that the response be proportionate to the threat, imminence and proportionality continue to bear on the assessment of the existence of the reasonableness of the belief (Hopkins & Easteal, 2010) and are likely “to continue to inform the way in which self-defence is applied in practice” (Law Reform Commission of Western Australia [LRCWA], 2007, p. 168). Therefore, to address barriers abused women often face in establishing their actions in self-defense, Victoria and Western Australia have both expanded the law of self-defense by introducing provisions expressly stating that imminence is not a legal requirement for self-defense to apply, *Crimes Act* 1958 (Vic) s322 M(1)(a); *Criminal Code Compilation Act* 1913 (WA) s248(4)(a).

To make self-defense more accessible for abused women, some jurisdictions have also introduced legislative guidance on the potential relevance of family violence evidence to explain how domestic abuse might have led the defendant to believe that their fatal violence was necessary and reasonable, even if the threat of harm was not immediate or the response proportional (Table 1). In Victoria, Western Australia, and South Australia, the provisions explicitly permit a range of social context evidence known to be important in understanding the dynamics of family violence, such as the history of the relationship and violence within it, the cumulative effect of family violence, and the possible consequences of separation from the abuser, *Crimes Act* 1958 (Vic) s322J(1); *Evidence Act* 1906 (WA) s38; *Evidence Act* 1929 (SA) s34W, while Queensland only makes admissible relevant evidence of the history of the domestic relationship, *Evidence Act* 1977 (Qld), s132B. Victoria, Western Australia, and South Australia have also introduced mandatory jury directions to ensure the jury is informed on the nature and dynamics of family violence and how it may be relevant to claims of self-defense, *Evidence Act* 1906 (WA) s39C, s39E, s39F; *Evidence Act* 1929 (SA) s34Y; *Jury Directions Act* 2015 (Vic) s59, s60. These new self-defense provisions work toward ensuring an abused woman's claim of self-defense will be properly assessed with reference to the wider social circumstances surrounding the offence and the cumulative impact of domestic violence.

### Specialized defense tailored to victims of abuse

Rather than expand the law of self-defense, Queensland is the only jurisdiction to have introduced a specialized partial defense tailored to “killing for preservation in an abusive domestic relationship,” *Criminal Code* 1899 (Qld) s304B. The new partial defense was designed to create a defense for “victims of domestic violence who, fearing for their lives, kill their abusers in circumstances that would otherwise constitute murder” (Edgely & Marchetti, 2011, p. 125), and unlike self-defense in Queensland,^1^ it may be available for those who kill in non-confrontational circumstances. However, while the defense eliminates the need for establishing an imminent threat, it has been criticized for operating as partial defense (Edgely & Marchetti, 2011; Hopkins & Easteal, 2010). This reduces a murder conviction to manslaughter, rather than a complete acquittal.

### Provocation and excessive self-defense

The controversial partial defense of provocation has also been subjected to substantial review and reform in both Australia and overseas (Fitz-Gibbon & Sheehy, 2019; Quick & Wells, 2012; Tyson, 2012). Provocation applies when a person has understandably lost control and responded with lethal force to provocative circumstances (Sheehy et al., 2012b). However, several jurisdictions have abolished the “outdated” defense in response to concerns that it operated in a gender-bias manner, inappropriately excusing men who killed their female partners out of anger, jealously, or possessiveness (Crofts & Tyson, 2013). In contrast, others expressed concerns for victims of prolonged abuse if provocation was abolished (Select Committee on the Partial Defence of Provocation [Select Committee], 2013; Tolmie, 2005). Instead, jurisdictions including Queensland, New South Wales, and Canada have retained provocation to ensure its continued availability for abused women who may not meet the requirements of self-defense but introduced provisions restricting its access for inappropriate male lethal violence (see Fitz-Gibbon & Sheehy, 2019).

Rather than provocation, some jurisdictions have re-introduced into legislation excessive self-defense to provide a more appropriate partial defense for abused women (Table 1). Excessive self-defense applies when a person believes that lethal force was necessary in self-defense, but that the belief was unreasonable in the circumstances (Sheehy et al., 2012b). The defense is intended to provide a more appropriate “halfway house” for abused women between a complete acquittal and conviction for murder, providing women with a “safety net” if claims of self-defense fail (LRCWA, 2007, p.180; VLRC, 2004, p. 99). However, many have argued that the defense is unnecessary because if an abused woman had a genuine belief in her need to use force, then she should be more adequately catered for under self-defense law (Fitz-Gibbon & Stubbs, 2012; Toole, 2012).

### Removal of partial defenses

While Victoria introduced their own version of excessive self-defense in 2005 with the creation of defensive homicide, a separate offence equivalent to manslaughter, it was abolished in 2014 in response to concerns that it had replaced provocation by excusing inappropriate male violence (Department of Justice, 2013; see also Naylor & Tyson, 2017). Instead, the Victorian government was of the view that self-defense should be the “primary focus” for women who kill in response to abuse (Department of Justice, 2013, p. 30). While some supported the decision to remove defensive homicide (Fitz-Gibbon, 2013), many expressed concern that its removal will disadvantaged those who kill in response to domestic abuse (King et al., 2016; McKenzie et al., 2014; Wake, 2015). With the abolishment of defensive homicide, Victoria joins jurisdictions like Tasmania and New Zealand that offer no partial defenses to homicide. In these jurisdictions, self-defense becomes an “all-or-nothing” venture, as women who are unsuccessful in raising self-defense will face a conviction for murder, with evidence to suggest that this may increase their risk of receiving a murder conviction when manslaughter may be more appropriate (Tolmie, 2015).

## Method

### Data Collection

To examine how abused women are currently treated in the Australian criminal justice system, this study collected and analyzed 69 cases of women prosecuted for killing an abusive male partner throughout Australia from 2010 to 2020.^2^ Multiple methods were adopted to identify relevant cases. This included searching through the legal databases AustLII and LexisNexis for reported and unreported legal decisions, web and database searches of media and newspaper articles, and other secondary sources, such as published research, coronial inquests, and government reports. All cases identified that involved a female killing a current or formal male partner (married, de facto, boyfriend, and sexual intimates) that mentioned domestic abuse were included in the study.

### Sample

Most of the cases occurred in Victoria (26%, *n =* 18), New South Wales (22%, *n =* 15), Western Australia (16%, *n =* 14), and Queensland (16%, *n =* 11), reflecting the larger populations in these jurisdictions. Less than 10% of cases occurred in the Northern Territory (7%, *n =* 5), South Australia (6%, *n =* 4), and Tasmania (3%, *n =* 2), while no cases identified occurred in the Australian Capital Territory. Consistent with previous research (Sheehy et al., 2012a), Indigenous women were over-represented in the sample, with 20 (29%) cases involving an Indigenous defendant, despite Indigenous persons constituting 3.3% of the total Australian population (Australian Bureau of Statistics, 2019). The age of the defendant at the time of offence was known in 66 (96%) cases, with ages ranging from 18 to 67 years old (*M *= 37, *SD *= 11.99).

### Data Coding and Analysis

Data for this study were extracted from a variety of available sources. Of the 69 cases identified, 52 (75%) had case law documents available (including 37 sentencing remarks, 16 appeal decisions, three trial verdicts),^3^ 18 (26%) were described in academic articles or government reports, four (6%) had a coronial inquest available, while information on nine (13%) cases was only available in media articles. Drawing upon the various sources of data, this study examined and coded each case for their (a) *legal outcomes*, including the original charge laid, the method of resolution (i.e., guilty plea or trial), the conviction received (if any), the basis of the conviction, and the sentenced received. The study also coded (b) *defendant characteristics* previously found to impact on the legal treatment of abused women (Douglas, 2012), such as their Indigenous status, whether they have a previous criminal history, and whether they had previously fought back. Lastly, the study also recorded (c) *case characteristics* likely to influence legal outcomes, including whether the case involved expert testimony or evidence, the immediate circumstances of the offence, the presence of alcohol or illicit drug use at the time of offence, and the method in which the defendant killed her abusive partner. In total, 21 variables were extracted from the data (see Appendix for coding dictionary).

The data were analyzed descriptively, examining the frequencies of the legal outcomes, defendant, and case characteristics. Using SPSS, a series of cross-tabulations were also performed to examine relationships between legal outcomes, defendant, and case characteristics. Cross-tabulations were also performed to compare jurisdictional differences, contrasting the jurisdiction in which the case was heard by legal outcomes, defendant, and case characteristics. Due to the comparatively small sample size and instances of missing data, inferential statistics were not employed.

## Results

### Legal Outcomes

Abused women continue to be predominately charged with murder. Of the 67 cases in which the original charged was known, 60 (90%) women were originally charged with murder, while seven were charged with manslaughter. However, Table 2 reveals that pleading guilty to manslaughter^4^ continues to be the most common legal outcome for abused women charged with homicide, with just under half (48%) of the cases resolved in this manner, while one case involved a defendant pleading guilty to the less serious offence of assault causing death.^5^ Consistent with previous research (Sheehy et al., 2012a), the majority (85%, *n =* 29) of defendants who pleaded guilty to manslaughter or a lesser offence did so for the prosecution to withdraw the murder charges against them. Just under half (49%, *n =* 34) of the defendants proceeded to trial, of which 15 (44%) were found guilty of manslaughter, seven (21%) were found guilty of murder,^6^ while 11 (16%) were acquitted and received no conviction. This was mostly on the basis of self-defense, while one was found to have not caused her partner's death after accidently striking him with her car as he was laying on the ground (*R v.* *Coman*, 2020).

**Table 2.** Legal Outcomes of Abused Women Homicide Prosecutions by Australian Jurisdiction (*n =* 69).

Legal outcome	Vic	NSW	WA	Qld	NT	SA	Tas	Total	%
Charges dropped							1	1	1
Guilty plea									
Lesser offence			1					1	1
Manslaughter	13	5	4	5	3	3		33	48
Trial									
Manslaughter	4	4	3	1	2	1		15	22
Murder		1	4	1			1	7	10
Acquitted	1	4	2	4				11	16
Quashed on appeal		1						1	1
Total	18	15	14	11	5	4	2	69	100

In total, 13 (19%) abused women received no conviction for their actions. This included one case in which a defendant had her manslaughter conviction quashed on appeal, with the New South Wales Court of Appeal finding that her conduct in arming herself with a knife was a reasonable response in the circumstances as she perceived it to be (*Silva v.* *R*, 2016). In another case, the female defendant had the charges against her dropped. Originally charged with murder for stabbing her violent partner three times following a prolonged physical assault in which he threatened to kill her, the accused was discharged from further proceedings after the prosecution concluded there was not enough evidence to disprove that she was acting in self-defense.

#### Jurisdictional differences

Table 2 reveals some discernible differences in how abused women were treated in each Australian jurisdiction. Although Victoria has made substantial changes to their homicide laws to improve access to self-defense, only one (5.5%) Victorian case resulted in an acquittal on the basis of self-defense. Instead, Victoria had the highest proportion (72%) of defendants pleading guilty to manslaughter, with only five (28%) women proceeding to trial. Following the removal of defensive homicide in 2014, this study identified seven cases that occurred in Victoria after this time, of which six were resolved by the defendant pleading guilty to manslaughter in exchange for the prosecution to withdraw murder charges against them. This finding may support concerns that the removal of partial defenses has negatively impacted on the legal position of abused women, as abused women may be under increased pressure to plead guilty to manslaughter rather than proceed to trial and risk a murder conviction. In contrast, the majority of defendants in New South Wales (67%, *n =* 10), Western Australia (64%, *n =* 9), and Queensland (54.5%, *n =* 6) proceeded to trial, with each jurisdiction continuing to retain at least one partial defense to homicide.

Queensland had the highest proportion of cases resulting in an acquittal (36%), followed by New South Wales (33%), with half of the abused women proceeding to trial in New South Wales being acquitted on the basis of self-defense. Although Queensland has not amended their self-defense laws, self-defense was successfully raised on three occasions, with one defendant successfully raising the defense for killing her abusive husband in a non-traditional self-defense scenario when the threat of harm was not immediate (*R v. Falls*, 2010). In this case, the defendant had been subjected to decades of serious physical and sexual violence from her husband, and following threats to kill her youngest child, sedated her husband and shot him in the head while he slept. Though Victoria and Western Australia introduced provisions explicitly stating imminence is not necessary for self-defense to apply, these provisions have not yet been utilized, and all other acquittals involved direct confrontations more consistent with traditional constructions of self-defense. Furthermore, Western Australia had the highest proportion (29%) of murder convictions, raising concerns over the legal position of abused women in this state.

#### Sentence received

All seven women found guilty of murder received a term of imprisonment. This included five defendants who received life imprisonment, while one woman in Tasmania was sentenced to 15 years imprisonment and the defendant found guilty of murder in New South Wales was sentenced to 36 years. Of the 49 women convicted of manslaughter or a lesser offence, the majority (94%, *n =* 46) received a term of imprisonment, while two (4%) received a fully suspended sentence, and one (2%) received a community corrections order. Of the women sentenced to a term of imprisonment, sentences ranged from 2.5 to 16 years, with an average sentence of 6.4 years (*SD = *2.6). While it may be expected that women who plead guilty would receive a more lenient sentence, there was no differences between the terms of imprisonment given to defendants who pleaded guilty to manslaughter (*M = *6.45, *SD *= 2.76) compared to those who were found guilty of manslaughter at trial (*M = *6.44, *SD *= 2.38). There were some differences between the length of imprisonment handed down for a manslaughter conviction by Australian jurisdiction: Western Australia had the lowest average sentence of four years imprisonment (*SD = *0.86), followed by South Australia with 5.25 years (*SD = *1.63) and New South Wales with 6.25 years (*SD *= 4.52). In Victoria and the Northern Territory, the average sentence was seven (*SD = *1.36) and 7.3 (*SD = *2.49) years, with this increasing to 8.16 years (*SD = *0.98) in Queensland.

#### Basis of conviction: partial defenses to homicide

Of the 69 cases identified, 48 (71%) resulted in a manslaughter conviction. The majority (60%, *n =* 29) of women convicted of manslaughter were convicted on the basis that they lacked the *mens rea* for murder, in that they lacked the intention to kill their abusive partner,^7^ with most (79%, *n =* 23) of these women pleading guilty to manslaughter on this basis (see Table 3). Over one-third (35%, *n =* 17) of the defendants were convicted on the basis of a partial defense to homicide, either Victoria's offence of defensive homicide (now repealed) (*n =* 4), Queensland's killing for preservation (*n =* 2), excessive self-defense (*n =* 3), provocation (*n =* 3), and diminished responsibility (or substantial impairment as it is known in New South Wales) (*n =* 5).

**Table 3.** The Basis of the Manslaughter Conviction and the Method of Resolution by Australian Jurisdiction (*n =* 46).

Jurisdiction	Lack of intent		Diminished responsibility^a^		Defensive homicide		Excessive self-defense		Provocation		Killing for preservation	
	Plea	Trial	Plea	Trial	Plea	Trial	Plea	Trial	Plea	Trial	Plea	Trial
Vic	11	2			2	2						
NSW	3	2	2	2								
WA	2	1					1	1				
Qld	3		1								1	1
NT	2	1							1	1		
SA	2						1			1		
Total	23	6	3	2	2	2	2	1	1	2	1	1

*Note.* Two cases in which basis of the manslaughter conviction was unknown are not included in this table.

^a^ Also known as substantial impairment in NSW.

In some instances, the continued availability of partial defenses appeared to have provided an appropriate alternative for abused women prosecuted for murder who did not fulfil the requirements for self-defense. For example, in the South Australian case of *DPP v. Narayan* (2011), the defendant set her abusive husband on fire after discovering he was having an affair with another woman. At her murder trial, the defendant argued that she only wanted to “purify” her husband with holy flame by burning a dot on his penis to keep him from leaving her, but lost control when he called her “a fat bitch” (Fewster, 2011). The jury acquitted her of murder but found her guilty of manslaughter on the basis of provocation. Despite being subjected to decades of physical and psychological abuse, she did not claim to act in self-defense, and without the continued availability of provocation, she may have been at risk of receiving a murder conviction.

However, the findings also support concerns that partial defenses have operated to undermine legitimate self-defense claims, with some of the defendants pleading guilty to a partial defense despite being subjected to a prolonged history of abuse and protecting themselves from an immediate attack at the time of the offence. For example, the defendant who pleaded guilty to Queensland's new preservation defense had experienced an extensive history of domestic violence and was physically assaulted immediately prior to the killing, including being grabbed by the throat, dragged along the ground, and urinated upon (*R v. Sweeney*, 2015). Following this attack, the defendant grabbed a knife to protect herself and stabbed her partner in the belief that she was at risk of death or serious injury. While the judge accepted her actions were out of fear and self-preservation in the context of an abusive relationship, such a finding in any other jurisdiction would have entitled the defendant to a complete acquittal on the basis of self-defense.^8^

### Defendant Characteristics

Table 4 shows the defendant characteristics of the abused women homicide cases. Nearly half (49%, *n =* 34) of the women were identified as having a history with substance abuse or dependence. Over a third (39%, *n =* 27) had a prior criminal history, with 20 (74%) of these women having a previous conviction for a violent offence. Just under half (49%, *n =* 34) of the cases had evidence that the female defendant had previously fought back. This included cases of mutual abuse in which both the male and female partner engaged in verbal or physical violence, as well as women who had previously used violence only in retaliation.

**Table 4.** Defendant Characteristics by the Legal Outcomes of Abused Women Homicide Prosecutions (*n =* 69).

Defendant characteristics	No conviction	Guilty plea		Found guilty		Total	%
		L/O	Mans.	Mans.	Murder		
Indigenous status							
Indigenous		1	12	5	2	20	29
Non-indigenous	13		21	10	5	49	71
History of substance abuse/dependence							
Yes	2		24	5	3	34	49
No	7		4	7	3	21	30
Unknown	4	1	5	3	1	14	20
Criminal history							
Yes	1		19	6	1	27	39
No	7		9	9	4	29	42
Unknown	5	1	5		2	13	19
Previously fought back							
Yes	2	1	22	6	3	34	49
No	10		8	8	3	29	42
Unknown	1		3	1	1	6	9

*Note.* L/O = offence less serious than manslaughter; Mans. = manslaughter/defensive homicide.

Consistent with previous research (Sheehy et al., 2012a; Stubbs & Tolmie, 2008), Indigenous defendants were more likely to plead guilty compared to non-Indigenous defendants (65% vs. 43%), with no Indigenous women being acquitted for their actions. Defendants with a history of substance abuse/dependence, criminal history, and previous evidence of violence appear less likely to have received no conviction compared to those with no substance abuse/dependence, no criminal history, and no history of violence. However, missing data and the small sample size prevents drawing strong conclusions regarding these findings. The number and percentage of cases involving Indigenous defendants differed across Australia: the Northern Territory had the highest proportion of Indigenous defendants (80%, *n =* 4), followed by Western Australia (43%, *n =* 6), New South Wales (27%, *n =* 4), Victoria (22%, *n =* 4), and Queensland (18%, *n =* 2). There were no Indigenous cases in South Australia and Tasmania. Missing data on the remaining defendant characteristics prevents finding any discernible differences between the jurisdictions.

### Case Characteristics

#### Expert evidence

Based on the information available, at least 12 (18%) women prosecuted for killing an abusive partner utilized expert evidence to support their defense,^9^ 31 (46%) entered psychiatrist and/or psychological reports during sentencing for mitigation, and 19 (28%) cases involved no expert evidence (see Table 5). Of the 34 abused women that proceeded to trial, just over one-quarter (26%, *n =* 9) utilized expert testimony, with five of these women being found guilty of manslaughter,^10^ while four were acquitted on the basis of self-defense. None of the women convicted of murder relied upon expert testimony, suggesting that abused women may be at risk of having their actions judged more harshly without expert evidence to support their defense.

**Table 5.** Expert Evidence by the Legal Outcomes of Abused Women Homicide Prosecutions (*n =* 68)*.*

Expert evidence	No conviction	Guilty plea		Found guilty			
		L/O	Mans.	Mans.	Murder	Total	%
Expert testimony, total	4		3	5		12	18
Traditional BWS	1		2	3			
Psych. assessment			1	1			
Expanded BWS	2						
Social context evidence	1			1			
Sentencing report, total	1^a^		21	7	2	31	46
Traditional BWS	1^a^		8	3			
Psych. assessment			13	4	2		
No expert evidence	7		5	2	5	19	28
Expert evidence unknown		1	4	1		6	9

*Note.* L/O = offence less serious than manslaughter; Mans. = manslaughter/defensive homicide; BWS = battered woman syndrome; Psych. assessment = Psychological assessment. The case in which the charges were dropped is excluded from the table.

^a^ In this case, the defendant was originally found guilty of manslaughter at trial and had a report entered in mitigation during sentencing. She later had her conviction overturned on appeal.

Most of the expert evidence introduced at trial or sentencing continued to be confined to forensic psychiatrists or psychologists undertaking psychological assessments focused on the defendant's pathology. This included *traditional BWS* approaches that explained the psychological impact of abuse or diagnosed the defendant with pathological conditions resulting from the abusive relationship (26%, *n =* 18), as well as *psychological assessments* that documented the domestic abuse or diagnosed the defendant with pathological conditions unrelated to the violence experienced (31%, *n =* 21).

In only four (6%) cases identified did the expert evidence take a more expansive approach that testified to the social context of the offending. This included two Queensland cases where psychiatrists testified under an *expansive BWS framework*. Although presenting evidence under BWS, the psychiatrists took an expansive approach that did not pathologize the defendant, but instead described the limited solutions available to women in leaving a violent relationship (*R v. Falls*, 2010), or explicitly explained that the concept of BWS is not a psychiatric diagnosis (*R v. Irsliger*, 2012) (see Douglas, 2012; Sheehy et al., 2014). In two cases, experts testified under the framework of *social context evidence.* This included a New South Wales case where a forensic psychiatrist testified to the cumulative impact of abuse, characterized the conduct of the deceased toward the defendant as torture, and explained how domestic abuse can be “almost impossible” to leave (*Stephen v. DPP*, 2018, para. 75). Lastly, only one case involved expert testimony provided by a family violence expert rather than a psychiatrist or psychologist (*DPP v. Williams*, 2014)*.* This case occurred in Victoria, with testimony provided by a law professor with 25-years’ experience in family violence that explained what it is like to live in an abusive relationship and addressing many commonly held misconceptions about domestic violence (King et al., 2016). In contrast, without legislative provisions explicitly allowing social context evidence to be admitted at the time, a case in Western Australia deemed similar social context evidence from a social worker inadmissible (*The State of Western Australia v. Liyanage*, 2016).

#### Circumstances of the offence

The majority of the defendants killed their abusive partner in confrontational circumstances, with nearly half (46%, *n =* 32) resorting to lethal violence either during or immediately following a physical assault, while over a third (36%, *n =* 25) killed their abusive partner during a verbal argument. Only 12 (17%) cases involved non-confrontational circumstances, with the women killing their partner when they were asleep or otherwise incapacitated, or by enlisting the assistance of others to commit the offence. Table 6 shows that the majority (77%, *n =* 10) of women who did not receive a conviction for their actions killed their partner during a physical confrontation, while women found guilty of murder most often (57%, *n =* 4) killed their partner in non-confrontational situations, and the majority (60%, *n =* 9) of women found guilty of manslaughter killed their partner during a verbal argument.

**Table 6.** Circumstances of the Offence by the Legal Outcomes of Abused Women Homicide Prosecutions (*n =* 69).

Circumstances of the offence	No conviction	Guilty plea		Found guilty			
		L/O	Mans.	Mans.	Murder	Total	%
Circumstances of the offence							
Physical confrontation	10		17	3	2	32	46
Verbal confrontation	2	1	12	9	1	25	36
Non-confrontational	1		4	3	4	12	17
Substance use at time of offence							
Yes	8	1	27	7	4	47	68
No	5		5	8	2	20	29
Unknown			1		1	2	3

*Note.* L/O = offence less serious than manslaughter; Mans. = manslaughter/defensive homicide.

As mentioned, Queensland was the only jurisdiction in which a defendant successfully raised self-defense in non-confrontational situations. In contrast, defendants who received no conviction in Victoria, New South Wales, Western Australia, and Tasmania killed their partners in traditional self-defense scenarios, resorting to lethal violence during or immediately following a physical assault, while one defendant acquitted in Western Australia killed her husband during a verbal argument. In such cases, the focus was on the immediate threat of harm at the time of offence, which did not require an understanding of the history of abuse to appreciate the women's response was reasonable in the circumstances. This focus was apparent in a Western Australian case where the defendant had been subjected to history of violence, but her defense lawyer stressed that her actions were not “an accumulation of domestic violence, it was sudden and uncharacteristic territory for her” (May, 2017).

While women who received no conviction were more likely to kill in a physical confrontation, the majority (53%, *n =* 17) of cases that involved a physical assault were resolved by a guilty plea to manslaughter. As many of these women were responding to an immediate threat of harm at the time of the offence, they could have been accommodated under the framework of self-defense had they raised the defense at trial. Take for example three cases resolved by a guilty plea to manslaughter in Western Australia. All had been charged with murder, and all had been defending themselves in response to an immediate attack from a partner that had been physically abusing them for years. In *The State of Western Australia v. Flett* (2012), the accused stabbed her long-term abusive partner after he lunged at her in a drunken rage and had only armed herself with a knife moments before the killing out of fear for her safety. In *The State of Western Australia v. Williams* (2011), the accused stabbed her partner in response to drunken assault while she was 14 weeks pregnant. She had been subjected to a substantial history of physical abuse by her partner, and the judge in this case accepted that the accused was afraid of his violence and that “her fear was fear with some real cause” (Banks, 2011). In *The State of Western Australia v. Byrne* (2015), the accused had stabbed her abusive partner while “defending herself from an alcohol fuelled attack,” with the judge acknowledging she had been subjected to a history of severe domestic violence, stating that “it was happenstance who was going to kill whom in this relationship” (Banks, 2015). Despite being subjected to a history of serious violence and protecting themselves from an immediate attack, by pleading guilty, these defendants potentially lost their chance of receiving an acquittal based on self-defense.

#### Alcohol and illicit drug use

Nearly two-thirds (68%, *n =* 47) of the cases involved the consumption of alcohol or illicit drugs at the time of the offence, with either the defendant (*n =* 7), the deceased (*n =* 13), or both (*n =* 27) drinking alcohol or using drugs prior to the offence. Table 6 shows that the majority (72%, *n =* 34) of cases involving substance use at the time of the offence resulted in a manslaughter conviction. There were some discernible differences between substance use at time of offence and jurisdiction, with Western Australia having the highest proportion of substance use (86%, *n =* 12), followed by the Northern Territory (80%, *n =* 4), Victoria (72%, *n =* 13), New South Wales (67%, *n =* 10), and Queensland (46%, *n =* 5).

Substance use was also frequently associated with cases involving a confrontational situation, with the majority of cases involving a physical (75%, *n =* 24) or verbal (68%, *n =* 17) confrontation involving alcohol or drug use at the time of the offence. Indeed, a common theme identified throughout the cases was that the defendants killed their abusive partner during a “drunken argument.” However, while many of these women were responding to an immediate threat of harm at the time of the offence, their actions were often attributed to alcohol or drug use in the context of an abusive relationship. For example, in the Victorian case of *R v.* *McLaughlin* (2016), the defendant pleaded guilty to manslaughter for killing her violent partner during a prolonged physical assault in which she had been hit, slapped, kicked, and strangled. During sentencing, the judge overlooked the objectively dangerous nature of the violence, and instead considered her actions to the be “the product of anger” in response to physical and emotional abuse (para. 11), noting that the couple's mutual drug abuse of methamphetamine contributed to their “volatile,” “turbulent,” and “violent” relationship (*R v.* *McLaughlin*, 2016, para. 2). Similar sentiments were expressed in the South Australian case of *R v. Collyer* (2012) where the defendant stabbed her partner while being physically attack, with the sentencing judge stating that the defendant had “difficulties with excessive consumption of alcohol” and that the couple's alcohol and drugs use was a “significant contributing factor” to their volatile and violent relationship.

#### Method of killing

Table 7 shows the legal outcomes by the method of how the women killed their abusive partner. Most of the women used a weapon (83%, *n =* 57) to kill their abusive partner, with the majority (70%, *n =* 48) using a knife or other sharp instrument to stab their partner. In these cases, the defendant rarely used an excessive level of violence required to cause the victim's death, with the majority (79%) using a knife to inflict only one or two stab wounds, while 10 (21%) stabbed their partner multiple times. In five (7%) cases, the defendants assaulted their partner with another weapon, such as a mallet, broomstick, or footstool, while four (6%) used a firearm to shoot their partner. Of the cases that did not involve a weapon, seven (10%) women enlisted the assistance of others to commit the offence, while three (4%) hit their partner with a car, and one (1%) set her partner on fire. In one case, the method of killing was unknown, as the body had been cut up with an electric saw and set on fire. There were no discernible differences between method of killing and jurisdiction, although most (*n =* 3) of the cases that involved a firearm occurred in Queensland.

**Table 7.** Method of Killing by the Legal Outcomes of Abused Women Homicide Prosecutions (*n =* 69).

Method of killing	No conviction	Guilty plea		Found guilty			
		L/O	Mans.	Mans.	Murder	Total	%
Stabbed one or two time	8	1	24	4	1	38	55
Stabbed multiple times	1		2	6	1	10	15
Others committed offence			2	2	3	7	10
Assaulted with weapon	1		2	2		5	7
Shot	2		1		1	4	6
Car	1		2			3	4
Fire				1		1	1
Unknown					1	1	1

*Note.* L/O = offence less serious than manslaughter; Mans. = manslaughter/defensive homicide.

## Discussion

Given the number of reforms that have been introduced in recent years aimed at improving legal responses to women who kill in the context of abuse, this study provides important insights into how the divergent law reforms have impacted on the legal position of abused women. The findings reveals legal barriers abused women continue to face in having their experience appropriately accommodated, highlighting important areas for future reform efforts.

### Continued Evidence of Overcharging

This study found that abused women continue to be predominately charged with murder, despite the prosecution being ultimately satisfied with a guilty plea to a lesser offence in most cases. This suggests that the prosecution are continuing to “overcharge” abused women with murder, which puts substantial pressure on them to plead guilty to manslaughter rather than risk a murder conviction at trial (see Stubbs & Tolmie, 2008). Consistent with previous research, many cases identified in this study where the abused woman pleaded guilty presented with strong defensive elements given the immediate threat of harm and history of abuse they had experienced (Sheehy et al., 2012a; Tyson et al., 2017). By pleading guilty, women with potentially legitimate claims of self-defense are ultimately forfeiting their right to a trial, potentially losing their chance at receiving an acquittal. This limits the intended impact of reforms introduced to improve access to defenses for abused women, as the new provisions and legal changes are not being adequately tested at trial. Had the defendants proceeded to trial and utilized the self-defense provisions available to them, some arguably may have had a realistic chance of receiving a complete acquittal. This was acknowledged by the sentencing judge in the Victorian case of *R v.* *Donker* (2018), who stated that given

the long history of domestic violence to which Ms Donker was subjected, the acts of violence against her the night before and the morning of the killing, and the provisions in the *Crimes Act* 1958 (Vic) concerning the admissibility of evidence of family violence and the directions which would be given to a jury on a trial of this nature about that evidence, had the matter gone to trial, Ms Donker would have had a sound basis to argue for an outright acquittal … (para. 99).

Reforms intended to improve access to defenses to homicide for abused women can only be effective if abused women proceed to trial and utilize the new provisions. As explained by Tolmie (2005), a consequence of plea negotiations means that “the case law on self-defence is not given the opportunity to develop so that it can accommodate the circumstances of battered defendants in an appropriate way” (p. 40). Furthermore, as plea negotiations are conducted in private, they are criticized for lacking accountability and transparency, limiting the ability to effectively evaluate the practical applications of law reforms (Flynn & Fitz-Gibbon, 2011; Stubbs & Tolmie, 2008).

Overall, to improve access to defenses to homicide for abused women, there needs to be increased attention toward the pre-trial stage of criminal proceedings and prosecutorial charging practices. Consistent with recommendations made previously, this paper provides support for prosecutorial guidelines strongly encouraging prosecutors to proceed to trial on manslaughter charges when there is strong evidence of self-defense (Select Committee, 2013, p. 168; VLRC, 2004, p. 110). As stated by Sheehy et al. (2012a), there must be “grave concerns about the integrity of a justice system in which the prosecution appears to be overcharging and then accepting guilty pleas in circumstances where there is evidence of self-defence” (p. 390).

### The Continued Need for Partial Defenses

Abused women continue to rely on a range of partial defenses to homicide that can accommodate a variety of different circumstances, both at trial and the basis of a plea, suggesting that partial defenses are still necessary for abused women homicide cases. Although some scholars argue that partial defenses detract away from considering an abused woman's actions as self-defense (Fitz-Gibbon, 2013), the high number of defendants pleading guilty in Victoria following the removal of defensive homicide supports concerns that abolishing partial defenses to homicide is “premature,” as self-defense remains not readily available for women who kill in the context of abuse (Tolmie, 2015, p. 658). Instead, abused women in Victoria appear to be under increased pressure to plead guilty to manslaughter as they no longer have a “safety net” to protect them from receiving a murder conviction at trial (McKenzie et al., 2014).

In contrast, jurisdictions that continue to offer partial defenses to homicide had a higher proportion of female defendants proceeding to trial. This may suggest that retaining partial defenses are needed to give abused women confidence to test their claims of self-defense at trial (LRCWA, 2007). Furthermore, in some instances, partial defenses captured cases that fell outside the scope of self-defense, supporting the viewpoint that retaining a range of defenses may more appropriate than attempting to create a “one-size-fits-all” model (Crofts & Tyson, 2013, p. 893; Wake, 2015, p. 152). However, it is important to note that some of the women convicted of a partial defense presented with strong defensive elements given the immediate threat of harm and prolonged history of abused they had experience, which would have supported a legitimate claim of self-defense and the chance at an acquittal. This support concerns that partial defenses have operated to undermine legitimate claims of self-defense (Fitz-Gibbon & Stubbs, 2012; Toole, 2013). As argued by Tarrant (2018), such manslaughter convictions avoid considering an abused woman's actions as self-defense, and while this reflects an “impulse for sympathy…sympathy is not justice” (pp. 219–220). Taken together, the findings from this study suggest a need for partial defenses to encourage abused women to go to trial; however, these defenses need to be applied cautiously with continued close monitoring to ensure they are applied as intended.

### Non-Confrontational Circumstances and Social Context Evidence

Despite law reforms aimed at improving access to self-defense for women who kill in non-traditional self-defense situations, these women are still infrequently relying on self-defense, with only one defendant successfully raised self-defense in non-confrontational circumstances (*R v. Falls*, 2010). Furthermore, this case occurred in Queensland, which has not amended their self-defense laws and continues to have strict legal requirements. In their analysis of this case, Sheehy et al. (2014) found that an expansive interpretation of self-defense combined with a progressive use of expert testimony supported the successful outcome. In contrast, despite Victoria and Western Australia both introducing provisions stating imminence is not necessary for self-defense to apply, these provisions were not utilized. Instead, the women who successfully raised self-defense in Victoria and Western Australia killed their abusive partner in response to an immediate threat of harm, fulfilling the traditional elements of self-defense. This finding is consistent with previous research that examined the impact of the 2005 Victorian reforms (Kirkwood et al., 2013; Tyson et al., 2017). As such, despite the “law on the books” providing greater access to self-defense (Braun, 2017, p. 1191), the expanded law of self-defense in legislation has still not been interpreted or applied in practice.

Western Australia's ability to accommodate an abused woman's claim of self-defense who kill in less traditional self-defense scenarios may have been limited by the lack of legislative guidance on the relevance of social context evidence at the time. As mentioned, in *The State of Western Australia v. Liyanage* (2016), social context evidence from a social worker was deemed as inadmissible, despite it demonstrating the objectively dangerous nature of domestic abuse and the high risk of serious harm that the accused had faced from her husband (Butler, 2016). In this case, the defendant unsuccessfully argued that she killed her sleeping husband in self-defense after being subjected to years of physical, emotional, and sexual abuse and coercing, controlling behavior, and was subsequently found guilty of manslaughter. Without the social worker's evidence, expert evidence presented at trial was limited to psychiatrists testifying under the traditional BWS framework. This pathologized her response to the prolonged abuse by focusing on her “cult-like mentality” and minimized the extent of the violence she had been subjected to (Tarrant et al., 2019, p. 41). Had the social context evidence been admitted, it could have more clearly conveyed “the extremity” of the defendant's reality of living within an abusive relationship (Butler, 2016, p. 341).

Under the new legislative changes introduced in 2020 in Western Australia, social context evidence known to be important in understanding the dynamics and cumulative impact of domestic violence is now explicitly allowed, *Evidence Act 1906* (WA) s38, and could have helped facilitate a better understanding of why her lethal defensive actions may have been reasonable and necessary response in the circumstances. This highlights the importance of legislating the admissibility of social context evidence to ensure such evidence is more widely accepted, supporting a broader range of expert witness testimony that can be used to better support abused women's claims of self-defense. As explained by Butler (2016), rules of evidence work to “filter and silence the stories of battered women at trial” (p. 321) and legislating social context evidence represent an important step in ensuring women's experience of violence is better understood and recognized within the legal system.

### Persistent Abused Women Stereotypes and Killing During a Drunken Argument

While several jurisdictions have changed their homicide laws based upon the assumption that women are more likely to kill an abusive partner in non-confrontational circumstances, only a small proportion of cases involved non-confrontational situations. Instead, most abused women resorted to lethal violence during a physical or verbal confrontation, with a common theme identified throughout many of the cases was that the women killed their abusive partner during a “drunken argument.” Despite many of these cases involving an immediate threat of harm fulfilling traditional self-defense scenarios, most of these women received a manslaughter conviction, with their defensive actions often attributed to substance abuse issues and a history of mutual violence within a “volatile” and “turbulent” relationship. However, attributing domestic violence to mutual dysfunction connected with alcohol abuse can overlook the objectively dangerous reality of living within an abusive relationship and the severity of the abuse perpetrated by the deceased (Wells, 2012).

This issue may be more pronounced for Indigenous defendants due to their structural and social disadvantage within Australian society and the combined effects of poverty, violence, alcohol and substance abuse, and gendered and racial discrimination that they may experience (Douglas et al., 2020; Stubbs & Tolmie, 2005). For instance, in *The State of Western Australia v. Byrne* (2015), the sentencing judge stated that domestic violence deaths in the Aboriginal community had become an “intractable social problem” and explained that he had often dealt with Indigenous women who had been the “victim of domestic abuse, usually where alcohol is involved, and have gone too far, frequently stabbing to death their partners, as you have done” (Banks, 2015). As such, while recent legal reforms have focused on increasing our understanding of nature and dynamics of domestic violence in situations when the threat of harm is not imminent, this is not the most common situation in which abused women kill. Instead, there needs to be an increased focus on addressing gender-based stereotypes and misconceptions of domestic violence that arise in cases involving Indigenous defendants, mutual violence, and substance abuse to ensure that the structural realities of women's lives are appropriately taken into account.

### Limitations and Future Research

As there is no comprehensive listing of homicide cases in Australia, this study was reliant on cases being publicly reported and accessible through legal and media databases. While numerous methods were adopted to identify relevant cases, not all cases are publicly reported or available, and the final sample of cases cannot claim to be exhaustive. However, as homicide cases are generally considered “newsworthy,” there is some likelihood that most of the cases in which women kill their partner would be recorded in some manner (Sheehy et al., 2012b, p. 485). The study was also limited due to its reliance on the information available in the secondary sources of data, which did not always provide the relevant information required. Future research could provide a more comprehensive understanding of these cases by interviewing legal professionals that have handled abused women's homicide cases. As pleading guilty to manslaughter continues to be the most common legal outcome among abused women's homicide cases, this research could focus on the pre-trial stage of criminal proceedings and the impact that plea negotiations may have on the resolution of such cases. Lastly, as this paper provided more of a quantitative overview of the current legal treatment of abuse women, future research could provide a more in-depth analysis of cases to provide further detail on how they were managed and represented within the legal system.

## Conclusion

While the legal reforms introduced in recent years represent an important first step in improving legal responses for abused women who kill, these changes need to be accompanied with other measures to ensure the new laws are interpreted and applied appropriately. As noted by Quilter (2011), law reforms rely “precariously upon the *practices* of those laws” (p. 26, emphasis in original). Law reform does not occur in a vacuum, and it is important to support the changes with specialized training and education for legal professionals to ensure that the laws are applied as intended and women's experiences of abuse are appropriately accommodated within the legal system. This could include educating legal professional on the persistent stereotypes and misconceptions of abused women and domestic violence, as well as how to effectively utilize different forms of expert evidence to best support abused women's claims of self-defense (Bradfield, 2002; Crofts & Tyson, 2013). With most cases resolved by a guilty plea, it is also important to educate the prosecution on the appropriate charges to lay in cases involving domestic abuse and ensuring that defense lawyers are aware of the relevant defenses available to most effectively represent their client. Just having the legislation in place does not mean that the law will be applied as intended, and there needs to be social and cultural change within the general community and legal system before we see a change in how women experience justice within criminal justice systems.

## Notes

1. Queensland is the only Australian jurisdiction to retain the requirement that defensive action be taken in response to a specific assault, *Criminal Code* 1899 (Qld) s271.

2. This period was selected to build on an earlier study conducted by Sheehy et al. (2012a) that analyzed legal outcomes of abused women’s homicide cases in Australia from 2000 to 2010.

3. There were also 11 types of other legal judgements, including evidence admissibility determinations, bail applications, applications for costs, and prasad directions.

4. In reporting the results, the term manslaughter also includes defendants that were convicted of defensive homicide, a separate offense that is equivalent to manslaughter.

5. Assault causing death, *Criminal Code Compilation Act 1913* (WA) s281, was introduced in Western Australia in 2008 to deal with the so called “one punch” homicide cases.

6. In one case, the Tasmanian Court of Appeal overturned the murder conviction and substituted the offence with being an accessory after the fact to murder, *Otto v Tasmania* (2021) TASCCA 15.

7. These included convictions based upon the commission of an unlawful and dangerous act, reckless manslaughter, and criminal negligence.

8. Similar circumstances were observed in other jurisdictions that retain partial defences to homicide (see, e.g., *R v. Collyer*, 2012; *The State of Western Australia v. Byrne,* 2015).

9. Expert evidence was used to support a defendant’s claim of self-defense (*n =* 6) as well as diminished responsibility/substantial impairment (*n =* 5) and manslaughter/defensive homicide (*n =* 1).

10. In two of these cases, the defendant failed to successfully argue self-defense, while three did not argue that their actions were in self-defense, instead arguing substantial impairment (*n =* 2) or manslaughter/defensive homicide (*n =* 1).
